# Factors associated with depression, anxiety, and stress symptoms among men in a rural area in Vietnam during COVID-19

**DOI:** 10.3389/fpsyt.2022.987686

**Published:** 2022-10-31

**Authors:** Van T. H. Hoang, Ha T. H. Nguyen

**Affiliations:** ^1^Department of Global Health, School of Preventive Medicine and Public Health, Hanoi Medical University, Hanoi, Vietnam; ^2^Department of Global Public Health, Karolinska Institutet, Solna, Sweden

**Keywords:** depression, anxiety, stress, rural area, COVID-19, men

## Abstract

**Background:**

The COVID-19 pandemic has affected health and well-being worldwide, and its psychological effects are receiving substantial attention in the scientific literature. Research to date shows that the pandemic has increased prevalences of depression, anxiety, and stress. This study aimed to estimate the prevalence of mental health symptoms and identify the associated factors among men in a rural area of Vietnam during the COVID-19 pandemic.

**Methods and findings:**

During July 15–31, 2020, we conducted a cross-sectional survey of 1,085 men from 18 years old in 11 rural districts in Thanh Hoa province, Vietnam, and assessed their mental health using the Depression, Anxiety and Stress Scale – 21 Items (DASS-21). Outcomes assessed were have a symptom of depression, anxiety, and stress; risk factors measured included age, religion, marital status, education, occupation, and financial status. Multiple linear regression was performed to determine the statistical significance of associations between risk factors and mental health symptoms. Findings showed that the prevalences of having a symptom of depression, anxiety and stress among participants were 6.39, 9.72, and 5.65%, respectively. Regression model showed being younger (95% CI: –0.030; –0.004, *p* = 0.001), men had high school degree (95% CI: –0.671; –0.074, *p* = 0.014), men living in nearly poor houshoulds (95% CI: 0.067, 1.905, *p* < 0.05) and poor housholds (95% CI: 0.608; 2.721, *p* < 0.05) had significantly lower depression scores than others.

**Conclusion:**

Prevalences of having symptoms of depression, anxiety and stress were much higher than in similar previous research in rural Vietnam, suggesting that mental health problems among men in this setting became more common during the COVID-19 pandemic. Age, religion, level of education and family income status were statistically significant predictors of mental health problems. These findings provide useful insights into the impact of pandemics on mental health.

## Introduction

Mental health can be defined as “a state of well-being in which the individual realizes his or her own abilities, can cope with the normal stresses of life, can work productively and fruitfully, and is able to make a contribution to his or her community” (1). Conversely, mental illness is a risk factor for several non-communicable and communicable diseases, and threatens maternal and child health (2). Mental disorders are responsible for 4.29% of disability-adjusted life years (DALYs), while depressive and anxiety disorders remained among the leading causes of burden worldwide (3); researchers have identified mental illness as a major risk factor for suicide (4–6), and described it as a “silent epidemic” that is not being addressed adequately in men (7). Several studies have revealed relationships between mental disorders and socio-demographic factors such as employment status, income, and poverty (8–10), associations that are typically stronger in men than women (11). The highest male suicide rates in Canada are in rural areas with high unemployment (12, 13). Depression, psychological discomfort, and common mental disorders were prevalent among males working in male-dominated businesses, according to a systematic review of depression in male-dominated industries and occupations (14).

The COVID-19 pandemic has proved to be a major cause of mental distress. The prevalence of depression symptoms among adults in the United States (US) during the pandemic (52.7%) was more than 3-fold higher than before it (24.7%) (15). Shi et al. reported that large minorities of Chinese people surveyed during the national COVID-19 lockdown period in early 2020 had symptoms of depression (27.9%), anxiety (31.6%), insomnia (29.2%) and acute stress (24.4%) (16). Adults in the United Kingdom aged 18 years and older, from low-income backgrounds and with few educational qualifications, were found to experience a similarly high rate of depression (17). Other research confirms that the prevalence of mental distress during the COVID-19 pandemic was not distributed evenly across populations: adults in the US with little income and savings had a high prevalence of depression (15), as did the unemployed in India (18). The impacts of the COVID-19 pandemic on depression, anxiety and stress have been found to vary by profession in both men and women (19).

Vietnam, which borders China, where the COVID-19 pandemic began (20), recorded its first case only 2 weeks after the World Health Organization warned about this new and deadly disease (20, 21). Due to a rapidly worsening epidemic, the decision of the first lockdown was imposed in Vietnam for 15 days from 1st April, 2020 (22). To 30 June 2020, Vietnam has gone through 75 days without COVID-19 cases in the community, among the 355 confirmed cases, no deaths (23); nonetheless, the restrictions on movement and interaction associated with COVID-19 left a dreadful impact on the everyday lives of many Vietnamese. Farmers faced a financial crisis due to the suspension of agricultural exports to China (24), and national COVID-19 restrictions caused a shortage of work and decrease in income, particularly for informal and low-skilled workers (25). Before the global pandemic, suicide accounted for more than 3,500 deaths in Vietnamese males for 2 years 2016 and 2017, double the number among females, and suicide ranked 8th among the leading causes of death (26). In Nguyen et al.’s study of suicide in rural areas of Vietnam, the proportion of unemployed among suicide attempters was 14%, double that in the general population (7.3%) (27). To date, no researchers have studied the mental health of men in rural areas of Vietnam during the COVID-19 pandemic. This study aimed to fill this gap by producing estimates of the prevalences of having depression, anxiety and stress symptoms and identifying associated factors among adult men living in rural Vietnam in mid-2020.

## Methods

### Study setting

We conducted a cross-sectional study in Thanh Hoa, a province in the North Central Coast region of Vietnam. The province’s population is about 3.6 millions, among 1.8 millions of males about 1.5 millions living in countryside (28). Its population is distributed unevenly – crowded in the midland and seaside delta and sparse in mountainous area. The major ethnic group in Thanh Hoa (and Vietnam as a whole) is Kinh. Nearly three quarters (71.8%) of the residents of Thanh Hoa work in agriculture, the remaining work in the industrial and service sectors (28).

### Study population

This study was part of a quality of life survey conducted by Hanoi Medical University. The sample size was calculated with the formula below:


$$n\geq \frac{Z_{1-\frac{\alpha^{\left(1-p\right)}}{2}}^{2}}{\varepsilon^{2}\,p}$$

where *n* = sample size; *Z* = *Z* statistic for a level of confidence of 95%; *p* = 0.08 (expected prevalence of the participants have depression) (29); α = type I error, in this case 0.05; ε = relative error, in this case 0.2. Therefore, the final sample size calculation was:


$$n\geq \frac{1.96^{2}\,\left(1-0.08\right)}{0.2^{2}\,0.08}=1105$$

Convenience sampling was performed in all 11 mountainous rural districts in Thanh Hoa province. We aimed to recruit 100 participants in each district. The final sample comprised 1,080 persons. The rate of response was 97.7%.

Protocol of mental health issue among men using data from quality of life survey in Thanh Hoa province were reviewed and approved by the School of Preventive Medicine and Public Health, Hanoi Medical University of Science Review Board on April 2, 2021.

First, in each district, two communes were randomized selected. Health care workers in each commune provided a list of eligible men living in the commune during study time. If a person was at least 18 years old, a resident of the area, and had no history of mental health problems in the past (according to data of health commune station), they were eligible. In each commune, we randomly selected 50 participants. After screening and verbal consent approval, trained study staff (medical students) administered a structured questionnaire during a face-to-face interview. The interviews took place in the house of participants for 30–45 mins.

### Data collection

Data was collected in July 2020, after the second wave of COVID-19 in Vietnam. Participants were asked to respond to a questionnaire consisting of two parts: sociodemographic information, and the Depression, Anxiety and Stress Scale – 21 items (DASS 21). Our trained students interviewed participants directly and entered the data on a Kobo toolbox form.

The sociodemographic information we collected included age, religion, ethnicity, marital status, level of education, occupation, and family financial status. Family financial status categories were based on government guidelines in 2016 (30).

The DASS 21 was used to obtain information on mental health symptoms. DASS 21 was developed by Lovibond and Lovibond in 1995 (31), and contains three self-report scales designed to measure depression, anxiety and stress; it should not be used for clinical diagnosis. In this study, we used the Vietnamese version of the DASS 21 translated and reviewed by the Vietnamese Committee on Anxiety/Panic Attack (32). Each of the three DASS 21 scales contains seven items, scored from 0 “did not apply to me at all – never” to 3 “applied to me very much, or most of the time – almost always”. The item scores are added to give scores for depression, anxiety, and stress ranging from 0 to 21 that can be categorized into three levels: normal, mild, moderate, severe, and extremely severe (Table 1) (33).

**TABLE 1:** Cut-off scores for conventional severity labels of depression, anxiety, and stress scale-21 items (DASS-21) scales.

	Depression	Anxiety	Stress
Normal	0–4	0–3	0–7
Mild	5–6	4–5	8–9
Moderate	7–10	6–7	10–12
Serve	11–13	8–9	13–16
Extremely severe	14+	10+	17+

### Statistical analysis

All statistical analyses were conducted with STATA software (STATA Corp., College Station, TX, United States). Descriptive analysis with measurements of frequency, mean, and standard deviation was used to express sociodemographic variables and results from the scales and questionnaires. The mean and standard deviation (SD) were calculated for the scores of anxiety, stress, and depression. Frequencies and percentages were calculated for the severity of DASS-21. Data were first analyzed using correlation separately for every three outcomes (stress, anxiety, and depression), separately. Multiple linear regression was used to test whether depression, anxiety and stress were significantly associated with sociodemographic variables. All demographic variables were inserted in three multiple linear regression models to test their relationship with stress, anxiety, and depression as dependent variables The statistical significance level was considered 0.05.

## Results

### Sociodemographic characteristics

Table 2 shows the numbers and percentages of our 1,080 participants by sociodemographic factors. About 70% of the participants were 18–44 years old, and most belonged to the Kinh ethnic group. Very few participants reported following a religion; most participants were married at the time of their participation. Nearly three quarters of participants reported high school education or higher. More than a quarter of participants were students, and over a tenth worked for government. The vast majority identified their family financial status as average, with less than 7% in the “nearly poor” and “poor” groups combined.

**TABLE 2:** Descriptive statistics of sociodemographic characteristics.

Factors	Participants	
	*n*	%
Overall	1080	100%
**Age (years)**		
Mean (SD)	35.50 (14.58)	
Median	31	
**Range**		
18–24	387	35.83%
25–44	378	35.00%
45–54	203	18.80%
55+	112	10.37%
**Ethnicity**		
Kinh	1,023	94.72%
Others	57	5.28%
**Religion**		
None	1,046	96.85%
Religious	34	3.15%
**Marital status**		
Single	456	42.22%
Married	609	56.29%
Divorced/Separated/Widowed/Other	15	1.39%
**Level of education**		
Less than high school	308	28.52%
High school or higher	770	71.30%
Other	2	0.19%
**Occupation**		
Medical worker	24	2.22%
Government staff	115	10.65%
Farmer	21	1.94%
Student	280	25.93%
Other	640	59.26%
**Family financial status**		
Rich	23	2.13%
Average	982	90.93%
Nearly poor	61	5.65%
Poor	12	1.20%

### Prevalence of depression, anxiety, and stress

**FIGURE 1:**
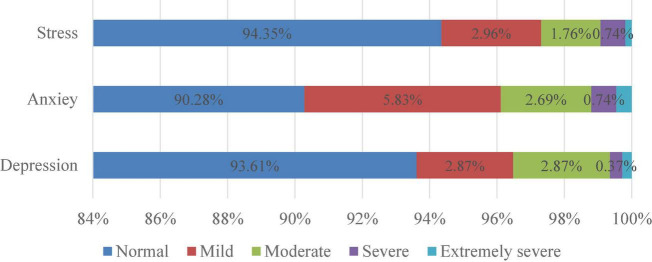
Levels of depression, anxiety, and stress among study participants.

**FIGURE 2:**
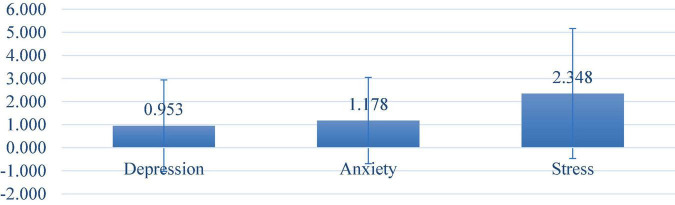
Mean score of depression, anxiety, and stress.

Abnormal levels of depression, anxiety and stress were observed in 6.39, 9.72, and 5.65% of the participants, respectively (Figure 1). The mean total score for participants was 0.95 (SD = 1.98) for depression, 1.18 (SD = 1.87) for anxiety, and 2.35 (SD = 2.82) for stress (Figure 2). Moderate scores for depression, anxiety, and stress were reported in 3.52, 3.89, and 2.69% of respondents, respectively.

### Mental health symptoms and associated factors

We performed multiple logistic regression analysis to identify the most important sociodemographic factors associated with depression, anxiety and stress among men in rural Thanh Hoa province. “Single” in marital status, “less than high school” in level of education, “medical worker” in occupation, and “rich” in family financial status were reference groups for each factor. For ethnicity and religion, the respective reference groups were “Kinh” and “none”.

Depression scores decreased significantly as participants’ age increased (Table 3). Participants who had a high school degree or a higher level of education had significantly lower depression scores than those who had not achieved a high school degree. Depression scores were lower among participants living in nearly poor and poor households.

**TABLE 3:** Multiple linear regression model for the prediction of depression.

Predictors	Univariate *r* (95%CI)	Multiple *r* (95%CI)
Age	–0.020 (–0.028, –0.012)	–**0.017 (**-0.030, 0.004)***
Ethnicity	0.433 (–0.097,.963)	0.363 (–0.163, 0.890)
Religion	0.134 (–0.545,.814)	0.114 (–0.556, 0.784)
**Marital status**		
Single	–0.113 (–1.125, 0.301)	–0.177 (–1.266, 0.912)
Married	–0.707 (–1.715, 0.301)	–0.403 (–1.427, 0.621)
Divorced/Separated/Widowed/Other	Ref.	Ref.
**Level of education**		
Less than high school	Ref.	Ref.
High school and higher	–0.023 (–0.286, 0.240)	–**0.373 (**–**0.672,** –0.074)**
Other	–0.471 (–3.238, 2.297)	–0.136 (–2.853, 2.853)
**Occupation**		
Medical worker	0.091 (–0.778, 0.960)	0.019 (–0.842 0.081)
Government staff	Ref.	Ref.
Farmer	–0.332 (–1.251, 0.588)	–0.466 (–1.403, 0.471)
Student	0.715 (0.286, 1.144)*	0.165 (–0.334, 0.664)
Other	0.262 (–0.130, 0.655)	0.126 (–0.275, 0.527)
**Family financial status**		
Rich	–0.776 (–1.590, 0.038)	–0.700 (–1.508, 0.107)
Average	Ref.	Ref.
Nearly poor	0.684 (0.174, 1.193)*	0.581 (0.067, 1.905)*
Poor	1.951 (0.912, 2.990)*	1.665 (0.608, 2.721)*

*p < 0.1, **p < 0.05, and ***p < 0.01.

Participants who reported a religion had significantly higher anxiety scores than those who did not, and family financial status was significantly and negatively associated with anxiety scores (Table 4). Participants who had at least a high school degree had significantly lower stress scores than those who did not, and participants with lower family financial status had higher stress scores than who lived in rich families (Table 5).

**TABLE 4:** Multiple linear regression model for the prediction of anxiety.

Predictors	Univariate *r* (95%CI)	Multiple *r* (95%CI)
Age	–0.001 (–0.009, 0.006)	0.006 (–0.006, 0.019)
Ethnicity	–0.219 (–0.718, 0.281)	–0.334 (–0.836, 0.169)
Religion	0.792 (0.154, 1.430)*	0.750 (0.111, 1.389)
**Marital status**		
Single	–0.608 (–1.570, 0.354)	–0.232 (–1.270, 0.806)
Married	–0.765 (–1.723, 0.193)	–0.402 (–1.379, 0.574)
Divorced/Separated/Widowed/Other	Ref.	Ref.
**Level of education**		
Less than high school	Ref.	Ref.
High school and higher	–0.436 (–0.291, 0.204)	–0.092 (–0.379, 0.193)
Other	–0.711 (–3.316, 1.894)	–0.771 (–3.362, 1.821)
**Occupation**		
Medical worker	–0.104 (–0.929, 0.720)	–0.086 (–0.907, 0.736)
Government staff	Ref.	Ref.
Farmer	–0.200 (–1.071, 0.672)	–0.456 (–1.350, 0.438)
Student	0.203 (–0.204, 0.610)	0.109 (–0.367, 0.584)
Other	0.047 (–0.325, 0.419)	–0.070, -0.453, 0.312)
**Family financial status**		
Rich	–**0.930 (**-**1.699,** -0.162)**	–**0.942 (**-**1.713,** -0.172)***
Average	Ref.	Ref.
Nearly poor	0.574 (0.093, 1.054)*	**0.588 (0.098, 1.078)**
Poor	**1.424 (0.443, 2.404)**	**1.289 (0.280, 2.295)**

*p < 0.1, **p < 0.05, and ***p < 0.01.

**TABLE 5:** Multiple linear regression model for the prediction of stress.

Predictors	Univariate *r* (95%CI)	Multiple *r* (95%CI)
Age	–0.004 (–0.015, 0.008)	0.004 (–0.014, 0.023)
Ethnicity	–0.262 (–1.015, 0.491)	–0.475 (–1.230, 0.280)
Religion	0.906 (–0.057, 1.869)	0.829 (–0.131, 1.789)
**Marital status**		
Single	–1.160 (–2.608, 0.288)	–0.626 (–2.186, 0.934)
Married	–**1.470 (**-**2.912,** -0.027)^**^	–1.050 (–4.944, 2.843)
Divorced/Separated/Widowed/Other	Ref.	Ref.
**Level of education**		
Less than high school	Ref.	Ref.
High school and higher	–0.333 (–0.706, 0.040)	–**0.539 (**-**0.967,** -1.111)*
Other	–1.088 (–5.009, 2.833)	–1.050 (–4.944, 2.843)
**Occupation**		
Medical worker	0.462 (–0.779, 1.702)	0.414 (–0.821, 1.648)
Government staff	Ref.	Ref.
Farmer	0.687 (–0.624, 2.000)	0.283 (–1.060, 1.626)
Student	0.489 (–0.123, 1.101)	0.256 (–0.458, 0.971)
Other	0.128 (–0.432, 0.688)	–0.156 (–0.730, 0.419)
**Family financial status**		
Rich	–**1.538 (**-**2.695,** -0.381)^**^	–**1.578 (**-**2.735,** -0.421)^**^
Average	Ref.	Ref.
Nearly poor	0.466 (–0.258, 1.190)	0.362 (–0.375, 1.098)
Poor	2.608 (1.131, 4.084)^**^	2.349 (0.835, 3.862)*

*p < 0.05 and ^**^p < 0.01.

## Discussion

The prevalences of depression, anxiety and stress in this study of men living in a rural area of Vietnam were 6.39, 9.72, and 5.65%, respectively. These results are considerably higher than the 3.9% prevalence of mental distress reported in a previous study (34), which in turn was four times the figure for the Vietnamese general adults population (3). The most compelling explanation for this difference is the impact of the COVID-19 pandemic and the related restrictions. The study was conducted after the second wave of the pandemic in Vietnam and a 2-week national social-distancing period. These factors have been proved to be associated with significantly higher prevalence of symptoms of mental illness worldwide (19, 35). However, compared to many countries, the prevalences of these symptoms in Vietnam are low. A recent systematic review and meta-analysis of Salari et al. indicated that prevalences of stress, anxiety and depression in general populations worldwide during the pandemic were 29.6, 31.9, and 33.7%, respectively (36). In Iraq, using the same DASS 21 questionnaire, researchers found 45.9% of participants reported at least some depression, 52.9% reported anxiety, and 17.5% of respondents suffered from stress of any severity (37). The relatively low rate of mental health symptoms during COVID-19 in Vietnam could be explained by the well-controlled situation nationwide. As of 1 July 2020, Vietnam had only 355 confirmed cases of COVID-19, no deaths, while the number of confirm cases globally was more than 10.3 millions with more than 500 thousands deaths, these number of the USA were more than 2.5 millions and more 120 thousands, respectively (23). Thanh Hoa had 18 COVID-19 patients, and all recovered after receiving treatment (38). Meanwhile, to that date there had been more than 10 million cases and 500,000 deaths globally, and 808,906 cases, 22,235 deaths in South East Asia (23). According to research on public trust in governments’ responses to COVID-19, Vietnam ranked highest rank among 45 countries surveyed, with 62% of Vietnamese respondents believing that their government was doing the “right amount” (39).

Age, religion, level of education, and family financial income were found to be significantly associated with the mental health of rural men in Thanh Hoa province during the COVID-19 pandemic. This finding aligns with Lorant et al.’s meta-analysis of socioeconomic status (SES) inequalities and depression, which indicated that individuals with low SES had higher odds of being depressed (40). Vietnam’s economic situation worsened during the pandemic; according to the Vietnam Economic Update Report, in the first quarter year 2020, “the growth rate was even lower than the worst-case scenario forecasted by the government” (41). In rural areas, at the end of June 2020, Vietnamese people reported a decrease in income of 38.3% from agricultural activities and 46.8% from non-farm activities (42). Regarding education, our results are similar to those of Araya et al., who found that people with less education had higher risk of common mental disorders (43). There is no clear explanation of the negative association between religion and stress in Vietnam; more research on this issue is needed. Our findings were consistent with those of some studies conducted in general populations that found rates of depression decreased as age increased (44, 45), but not with the results of Giang et al., who found that rural Vietnamese men aged 45–60 had significantly higher odds of mental distress than men aged 18–24, this study was conducted in a rural district of Hanoi, Vietnam (34). More research on the association between age and mental health of rural men is needed.

The strengths of this study include its participants (adult male), study site (rural area), and the special study period. However, our study has some limitations. First, this was a cross-sectional study and used convenience sampling, so we cannot draw conclusions about causal relationships between sociodemographic characteristics and mental health symptoms. Second, DASS 21 is a self-report measurement and may not reflect clinical diagnoses of mental illnesses.

## Conclusion

During the COVID-19 pandemic, mental health symptoms were more prevalent among rural men in Thanh Hoa province, Vietnam, than in previous research. Factors associated with depression, anxiety and stress symptoms were age, level of education, and family income status (negative) and religion (positive). Therefore, mental disorder prevention should target young adult males who have low level of education and poor family financial status. Future research should focus on the association between age and mental health problem in moutainous rural area as well as on identifying and addressing the proximal and distal socioeconomic factors that influence men’s mental health and exploring the numerous social connections and support systems that men regularly engage in to improve their mental health and wellbeing.

## Data availability statement

The raw data supporting the conclusions of this article will be made available by the authors, without undue reservation.

## Ethics statement

The studies involving human participants were reviewed and approved by Science Research Committee of Institute for Preventive Medicine and Public Health, Hanoi Medical University. Written informed consent for participation was not required for this study in accordance with the national legislation and the institutional requirements.

## Author contributions

VH was responsible for the study design, data collection, manuscript preparation, and revision. HN performed statistical analysis and wrote the first draft of the manuscript. Both authors contributed to the article and approved the submitted version.

## Abbreviations

DASS-21: depression, anxiety, and stress scale-21 items.
